# Gated Dehazing Network via Least Square Adversarial Learning

**DOI:** 10.3390/s20216311

**Published:** 2020-11-05

**Authors:** Eunjae Ha, Joongchol Shin, Joonki Paik

**Affiliations:** Department of Image, Chung-Ang University, Seoul 06974, Korea; eunjae_ha@ipis.cau.ac.kr (E.H.); jcshin@ipis.cau.ac.kr (J.S.)

**Keywords:** haze removal, generative adversarial network, gated structure

## Abstract

In a hazy environment, visibility is reduced and objects are difficult to identify. For this reason, many dehazing techniques have been proposed to remove the haze. Especially, in the case of the atmospheric scattering model estimation-based method, there is a problem of distortion when inaccurate models are estimated. We present a novel residual-based dehazing network model to overcome the performance limitation in an atmospheric scattering model-based method. More specifically, the proposed model adopted the gate fusion network that generates the dehazed results using a residual operator. To further reduce the divergence between the clean and dehazed images, the proposed discriminator distinguishes dehazed results and clean images, and then reduces the statistical difference via adversarial learning. To verify each element of the proposed model, we hierarchically performed the haze removal process in an ablation study. Experimental results show that the proposed method outperformed state-of-the-art approaches in terms of peak signal-to-noise ratio (PSNR), structural similarity index measure (SSIM), international commission on illumination cie delta e 2000 (CIEDE2000), and mean squared error (MSE). It also gives subjectively high-quality images without color distortion or undesired artifacts for both synthetic and real-world hazy images.

## 1. Introduction

Outdoor images are degraded by various atmospheric particles such as haze and dust. Especially, haze reduces the visibility of the image and disturbs the clarity of distant objects because of the effect of light scattering by particles in the air. Early dehazing techniques were based on mathematical optimization. Huang et al. proposed a visibility restoration (VR) technique using color correlation based on the gray world assumption and transmission map based on depth estimation [1]. Tan et al. proposed a Markov random field-based graph cut and belief propagation method to remove haze without using geometrical information [2]. Ancuti et al. removed the haze by identifying the hazy region through hue disparity between the original image and the ‘semi-inverse image’ created by applying a single per pixel operation to the original image [3]. Shin et al. removed the haze using a radiance and reflectance combined model and structure-guided $l_{0}$ filter [4]. Qu et al. presented a dehazing method based on a local consistent Markov random field framework [5]. Meng et al. presented the $l{}_{1}$ norm-based contextual regularization and boundary constraints-based dehazing method [6]. Liang et al. proposed a generalized polarimetirc dehazing method via low-pass filtering [7]. Hajjami et al. improved the estimation of the transmission and the atmospheric light applying to Laplacian and Gaussian pyramids to combine all the relevant information [8].

In spite of the mathematical beauty, a mathematical optimization-based method cannot fully use the physical property of the haze. To solve that problem, various physical model-based dehazing methods were proposed. He et al. estimated the transmission map by defining the dark channel prior(DCP), which analyzed the relationship between the clean and the hazy images [9]. Zhu et al. proposed a method of modeling the scene depth of the hazy image using color attenuation prior (CAP) [10]. Bui et al. calculated the transmission map through the color ellipsoid prior applicable in the RGB space to maximize the contrast of the dehazed pixel without over-saturation [11]. Tang et al. proposed a learning framework [12] by combining multi-scale DCP [9], multi-scale local contrast maximization [2], local saturation maximization, and hue disparity between the original image and the semi-inverse image [3]. Dong et al. used a clean-region flag to measure the degree of clean-region in images based on DCP [13].

**Figure 1:**
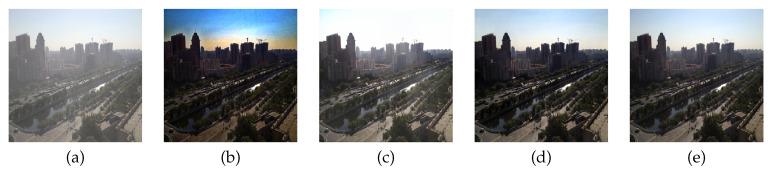
Comparison between the proposed method and the atmospheric scattering model-based methods: (**a**) input image, (**b**) DCP [9], (**c**) DCPDN [17], (**d**) the proposed method, and (**e**) the clean image.

However, these methods can lead to undesired results when the estimated transmission map or atmospheric light is inaccurate. Recently, deep learning-based transmission map estimation methods were proposed in the literature. Cai et al. proposed a deep learning model to remove the haze by estimating the medium transmission map through the end-to-end network, and applied it to the atmosphere scattering model [14]. To estimate the transmission more accurately, Ren et al. provided a multi-scale convolutional neural network (MSCNN) [15], and trained the MSCNN using the NYU depth and image dataset [16]. Zhang et al. presented a densely connected pyramid dehazing network (DCPDN), and reduced the statistical divergence between real-radiance and estimated result using the adversarial networks [17]. If the weight parameters, which reflect the transmission map and atmospheric light, are inaccurately learned, the DCPDN results in a degraded image. Figure 1b,c show image dehazing results based on two different atmospheric scattering models [9,17], respectively. As shown in the figures, inaccurate estimation results in over-saturation or color distortion since the transmission map is related to the depth map of the image. On the other hand, the proposed method shows an improved result without saturation or color distortion as shown in Figure 1d, which is very close to the original clean image shown in Figure 1e.

As previously discussed, atmospheric scattering model-based dehazing methods result in undesired artifacts when the transmission map or atmospheric light is inaccurately estimated. To solve this problem, Ren et al. proposed a dehazing method that fuses multiple images derived from hazy input [18]. Qu et al. proposed a pixel-to-pixel dehazing network that avoids the image to image translation by adding multiple-scale pyramid pooling blocks [19]. Liu et al. observed that the atmosphere scattering model is not necessary for haze removal by comparing direct and indirect estimation results [20]. Inspired by these approaches, we present a novel residual-based dehazing network without estimating the transmission map or atmospheric light. The proposed dehazing generator has an encoder–decoder structure. More specially, in the proposed encoder–decoder network, the local residual operation and gated block can maximize the receptive field without the bottleneck problem [18]. In addition, to reduce the statistical difference between the clean and dehazing result, the proposed discriminator decides if the generator’s result is real or fake by comparing with the ground truth. The proposed network is trained by minimizing the $l{}_{1}$-based total variation and Pearson $X^{2}$ divergence [21,22]. This paper is organized as follows. In Section 2, the related works are presented. The proposed gated dehazing network is described in Section 3 followed by experimental results in Section 4, and Section 5 concludes this paper with some discussion.

## 2. Related Works

This section briefly surveys existing dehazing methods that partly inspired the proposed method. In particular, we describe: (i) the formal definition of the atmosphere scattering model, (ii) how the gated operation is used not only in the deep learning model but also in dehazing, and (iii) how the generative adversarial network (GAN) is used in a dehazing method.

### 2.1. Atmosphere Scattering Model

A haze image $I\left(x\right)$ acquired by a digital image sensor includes ambiguous color information due to the scattered atmospheric light *A* whose effect is proportional to the distance from the sensor $d\left(x\right)$. To estimate the scene radiance of the haze image, the atmospheric scattering model is defined as [23]

(1) $$I\left(x\right)=J\left(x\right)e^{-\beta d\left(x\right)}+A\left(1-e^{-\beta d\left(x\right)}\right),$$

where *x* represents the pixel coordinates, $I\left(x\right)$ the observed hazy image, $J\left(x\right)$ the clean haze-free image, *A* the atmospheric light, $e^{-\beta d\left(x\right)}=t\left(x\right)$ the light transmission, $d\left(x\right)$ the depths map of the image, and β the scattering coefficient.

### 2.2. Gated Network

Ren et al. proposed a gated fusion network (GFN) that learns a weight map to combine multiple input images into one by keeping the most significant features of them [18]. In their original work, they referred to the weight map as the confidence map. The weight map in a GFN can be represented as [18,24,25]

(2) $$w_{1},w_{2},\cdots ,w_{n}=Gate\left(F_{1},F_{2},\cdots ,F_{n}\right),$$

where $F_{i}$ for i=1,…,n represents the feature map of the *i*-th layer and $w_{i}$ for i=1,…,n, the weight or confidence map through the gate. Using $F_{i}$ and $w_{i}$, the final feature is computed as

(3) $$F_{t}=w_{1}⊙F_{1}+w_{2}⊙F_{2}+\ldots +w_{n}⊙F_{n},$$

where ⊙ represents the element-wise multiplication. Chen et al. showed that the gated operation-based haze removal method is effective through the smooth dilated convolution with a wide receptive field and the gated fusion method using elemental-wise operation [24].

### 2.3. Generative Adversarial Network

The GAN is learned by repeating the process of generating a realistic image in which the generator tries to confuse the discriminator. GAN is formulated as [22]

(4) $$\arg\min_{G}\min_{D}V\left(D,G\right)=E_{x∼p_{data}\left(x\right)}\left[\log D(x)\right]+E_{z∼p_{z}\left(z\right)}\left[\log\left(1-D\left(G(z)\right)\right)\right],$$

where *x* and *z* respectively represent the real image and random noise, and *D* and *G* respectively the discriminator and generator [22]. When the random noise *z* is replaced to haze image *I*, this adversarial learning can be effectively applied to dehazing approach [17].

## 3. Proposed Method

**Figure 2:**
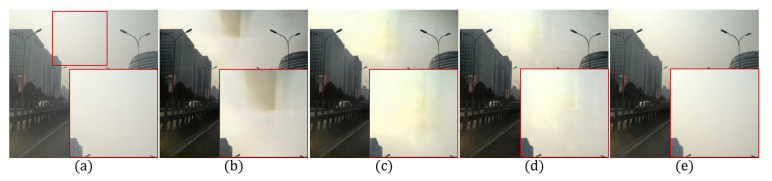
Effects of network structure: (**a**) hazy input image, (**b**) without residual blocks, (**c**) without gate block, (**d**) the proposed method, (**e**) the clean image.

Since it is hard to estimate the accurate transmission in Equation (1), we propose a novel residual-based dehazing method that does not use the transmission map. Figure 2 shows the effect of structures to the network. This section describes details of the proposed method including gate network, global residual, and adversarial learning.

### 3.1. The Gated Dehazing Network

**Figure 3:**
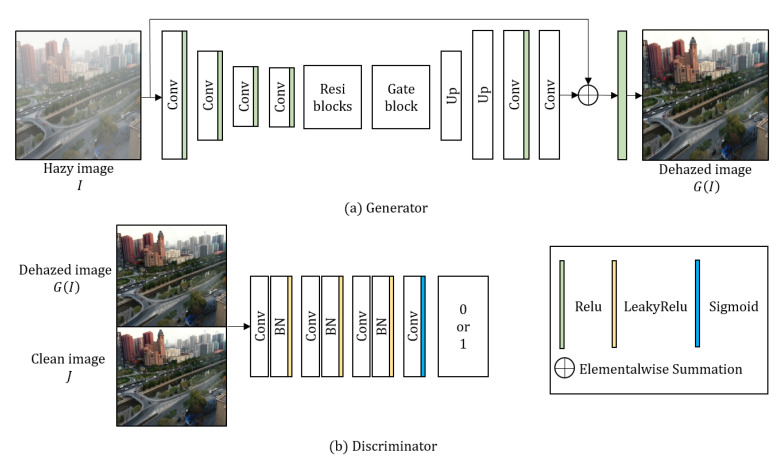
Overall architecture of the proposed network.

To remove the haze, the proposed generator takes a single haze image as an input as shown in Figure 3a. The generator goes through the processes of encoder, residual blocks, gate block, decoder, and global residual operation. In the encoding process, the resolution of the input image is reduced twice through convolution layers, while the number of output channels accordingly increases. The encoding process of proposed method can be expressed as

(5) $$E_{k}=\sigma_{k}\left(\left(\cdots \sigma_{1}\left(\left(W_{1}*I\right)+b_{1}\right)\cdots \right)*W_{k}+b_{k}\right),$$

where $W_{k},b_{k}$, and $\sigma_{k}$ respectively represent the weight, bias, and Relu activation function [26] of the *k*-th convolutional layer, and * the convolution operator. The hazy image *I* was used as an input in the encoding process to have 3 input channels. The following convolutional layers have 64 output channels with 3×3 kernel. To extract feature maps, the input image is reduced by 4 times using stride 2 twice. In addition, 128 output channels and K=4 layers are used in the encoding block. Since the encoded features have low-scale and large channels, the proposed network has large receptive fields. In other words, the proposed network computes more wider context information [27]. However, the bottleneck effect decreases the performance of the proposed network because too many parameters are required to restore the features with large receptive fields [28]. If a network is constructed without residual blocks, the bottleneck problem is as shown in Figure 2b.

To solve this problem, the proposed network also uses the hierarchical information from low-level to high-level features using five residual blocks as

(6) $$\begin{array}{c} R_{1}=E_{4}+\sigma_{4}^{e}\left(\left(W_{4}^{e}*E_{4}\right)+b_{4}^{e}\right),\\ R_{n}=\sigma_{n}^{r}\left(R_{n-1}+\sigma_{n-1}^{r}\left(\left(W_{n-1}^{r}*R_{n-1}\right)+b_{n-1}^{r}\right)\right),2\leq n\leq 5 \end{array}$$

where $W_{4}^{e},E_{4},b_{4}^{e}$, and $\sigma_{4}^{e}$ respectively represent the weight, feature map, bias, and Relu activation function for the last layer of the previous encoding block. $R_{1}$ represents the first residual feature map through element-wise summation with the last feature map of the encoding block. $W_{n-1}^{r}$, $R_{n-1}$, $b_{n-1}^{r}$ and $\sigma_{n-1}^{r}$ respectively represent the weight, residual feature map, bias, and Relu activation function of the n−1th block of the residual process. For the residual blocks of the proposed method, 25 layers were used.

Figure 2c shows that the bottleneck problem is solved by adding residual blocks. However, the enlarged red box does not converge due to the hierarchical information generated from the residual blocks. To solve this problem, we obtain the weights from low to high level through the gating operation inspired by the GFN [18] for the feature map to contain a hierarchical information generated in the residual blocks. The gating operation to obtain the feature map through the element-wise multiplication of the acquired weights and the value generated in the residual blocks can be defined as

(7) $$\begin{array}{c} G_{f_{1,2,\cdots ,5}}=\left(W_{1}^{gate}*\left[R_{1},R_{2},\cdots ,R_{5}\right]+b_{1}^{gate}\right),\\ G_{f_{t}}=\sum_{k=1}^{5}G_{f_{k}}\circ R_{k}, \end{array}$$

where $W_{1}^{gate}$ and $b_{1}^{gate}$ respectively represent the weight and bias of gate blocks. “$\left[\cdot \right]$” represents the concatenation operation of residual feature maps with hierarchical information from low to high level, and “∘” the element-wise multiplication of $G_{f_{1,2,\cdots 5}}$ and hierarchical feature maps $R_{1,2,\cdots 5}$.

The decoding layer reconstructs the restored image based on the generated and computed features [29]. In the decoding process, the resolution is restored as

(8) $$D_{up}=\sigma_{1}^{d}\left(W_{2}^{d}*\left(\left(W_{1}^{d}*\left(G_{f_{t}}{↑}_{2}\right)+b_{1}^{d}\right){↑}_{2}\right)+b_{2}^{d}\right),$$

where $W_{n}^{d},b_{n}^{d}$, and $\sigma_{1}^{d}$ respectively represent weight, bias, and Relu activation function in the decoding layer, and “${↑}_{2}$” the up-sampling operation with a scale of 2. The proposed decoding layer repeats the 3×3 convolution after up-sampling using bilinear interpolation twice to decode the image to the original resolution.

The global residual operation can effectively restore degraded images, and can improve the robustness of the network [30,31]. In this context, we can formulate the relationship between the global residual operation and the input hazy image *I* as

(9) $$G\left(I\right)=\sigma_{1}^{gr}\left(D_{up}+I\right),$$

where $\sigma_{1}^{gr}$ represents the Relu activation function in the global residual operation. Through summation of decoded $D_{up}$ and input hazy image *I*, the generator’s dehazed image $G\left(I\right)$ is acquired. We designed the network structure to solve the bottleneck problem for clean results shown in Figure 2d, where the proposed gated network can generate more hierarchical features. A list of parameters of the generator are given in Table 1 and Table 2.

Although it was successful to obtain enhanced results of synthetic data using only a generator, adversarial learning was applied to obtain more robust results in real-world images. For the adversarial learning, we also propose the discriminator inspired by [32], which increases the number of filters while passing through layers and has a wider receptive field. The discriminator takes the dehazed image $G\left(I\right)$ or clean image *J* as input. To classify the images, the proposed discriminator estimates the features using four convolutional layers as

(10) $$\begin{array}{c} D\left(G\left(I\right)\right)=\Phi \left(\Psi_{4}^{d}\left(BN_{4}^{d}\left(\cdots \Psi_{1}^{d}\left(BN_{1}^{d}\left(G\left(I\right)*W_{1}^{d}+b_{1}^{d}\right)\right)\cdots \right)*W_{4}^{d}+b_{4}^{d}\right)\right),\\ D\left(J\right)=\Phi \left(\Psi_{4}^{d}\left(BN_{4}^{d}\left(\cdots \Psi_{1}^{d}\left(BN_{1}^{d}\left(J*W_{1}^{d}+b_{1}^{d}\right)\right)\cdots \right)*W_{4}^{d}+b_{4}^{d}\right)\right), \end{array}$$

where $W_{n}^{d},b_{n}^{d},BN_{n}^{d},\Psi_{n}^{d}$, and Φ respectively represent the *n*-th weight, bias, batch normalization, leaky Relu function [33], and sigmoid function in the discriminator. As in Equation (10), the discriminator takes $G\left(I\right)$ or *J* as input, and the input channel is set to 3. As the number of channels passed through the layer increases, 192 output feature maps were extracted. In the last layer, the discriminator extracts a single output feature map to classify the image, and determines whether it is valid (1) or fake (0) applying to sigmoid function. Detailed parameters of the discriminator are given in Table 3.

### 3.2. Overall Loss Function

The loss function of the proposed method consists of $L_{l_{1}}$, $L_{vgg}$, $L_{adv(D)}$, and $L_{adv(G)}$. $L_{l_{1}}$ can be obtained by using the mean absolute error between the dehazed image $G\left(I\right)$ and the generated clean image *J*, which is formulated as

(11) $$L_{l_{1}}=\frac{1}{M}{∥G\left(I\right)-J∥}_{1},$$

where M=H×W×C represents the size of input image. $L_{vgg}$ used a pre-trained VGG16 network that can extract perceptual information from images and enhance contrast [21]. The equation for obtaining the VGG16 loss can be formulated as

(12) $$L_{vgg}=\sum_{k=1}^{4}\frac{1}{N_{k}}{∥V_{k}\left(G\left(I\right)\right)-V_{k}\left(J\right)∥}_{2}^{2},$$

where $V_{k}\left(\cdot \right)$ represents the pre-trained VGG16 network, and k=1,⋯,4 the number of layers containing important features. $N_{k}$ is the product of the height, width, and channel of the VGG16 layer corresponding to *k*.

For stable learning and higher quality outputs, least squares generative adversarial network (LSGAN) [34], which is an improved version of the original GAN, was applied to our proposed model. The generator creates a dehazed image $G\left(I\right)$, and the discriminator distinguishes whether the dehazed image is real or fake. The resulting adversarial loss is calculated as

(13) $$\begin{array}{c} \min_{D}adv\left(D\right)=\lambda_{D}\left(\frac{1}{2}E_{J∼p_{D}\left(J\right)}\left[{\left(D(J)-1\right)}^{2}\right]+\frac{1}{2}E_{I∼p_{G}\left(I\right)}\left[{\left(D(G(I))\right)}^{2}\right]\right),\\ \min_{G}adv\left(G\right)=\lambda_{G}\left(\frac{1}{2}E_{I∼p_{G}\left(I\right)}\left[{\left(D(G(I))-1\right)}^{2}\right]\right), \end{array}$$

where hyper-parameters $\lambda_{D},\lambda_{G}=0.2$ were used for the discriminator and generator losses, respectively. As shown in Equation (13), the proposed adversarial loss goes through an optimization process that minimizes the Euclidean distance between the discriminator and generator.

### 3.3. Training Details

**Figure 4:**
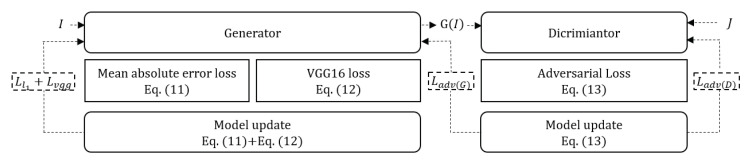
Learning process.

Figure 4 shows the learning process of the proposed model including: (i) computing the mean absolute error and perceptual losses using Equations (11) and (12), (ii) updating the generator after adding the previously obtained losses, and (iii) updating the discriminator loss in Equation (13). The generator loss is finally updated.

## 4. Experimental Results

To train the proposed model, Adam optimizer was used with learning rate ${\left(10\right)}^{-4}$ for combined $L_{l_{1}}$ and VGG16 loss $\left(L_{l_{1}}+L_{vgg}\right)$ and ${\left(10\right)}^{-6}$ for adversarial loss $\left(L_{adv(G)},L_{adv(D)}\right)$. We used 10,000 images from indoor training set (ITS) and 18,200 images from outdoor training set (OTS) Part 1 for the training dataset [35]. We implement our model using a personal computer with a 3.70 GHz Intel Core i9-9900K processor and NVIDIA GTX 2080ti 12GB GPU. Training time took almost 30 hours and Pytorch was used as the framework. In consideration of the training speed and stability, the batch size was set to 10, and every image was resized to 256×256 for the input patch of the network. For testing, an input image was resized to 512×512 to generate the corresponding output. We used 500 synthetic objective testing set (SOTS) outdoor and indoor images, and proved the dehazing performance of the proposed model for 500 real-world hazy images provided by fog aware density evaluator (FADE) [36]. For a fair comparison, state-of-the-art dehazing methods including: DCP [9], CAP [10], radiance-reflectance optimization (RRO) [4], all-in one network (AOD) [37], DCPDN [17], and GFN [18] were tested together with the proposed method.

### 4.1. Performance Evaluation Using Synthetic Data

To synthesize a hazy image, depth map information is required. Li et al. synthesized hazy images using both indoor and outdoor data with depth information [35]. For example, in an outdoor environment, the depth range extends up to several kilometers, whereas in an indoor environment, it has several meters. For this reason, we used 500 SOTS outdoor and indoor images for experiments with various depths [35] and synthetically simulated haze images to evaluate the objective performance in the sense of MSE and PSNR, SSIM [38], and CIEDE2000 that calculates the color difference in the CIELAB space [39]. If two images become closer, SSIM approaches 1, while CIEDE2000 approaches 0.

Table 4, Table 5, Table 6 and Table 7 show the averages of quantitative evaluation results for 500 SOTS outdoor and indoor images with consideration of statistical significance. As shown in Table 4, Table 5, Table 6 and Table 7, the proposed method shows higher performance than any other methods in the sense of all metrics.

### 4.2. Similarity Comparison Using Benchmarking Dataset

**Figure 5:**
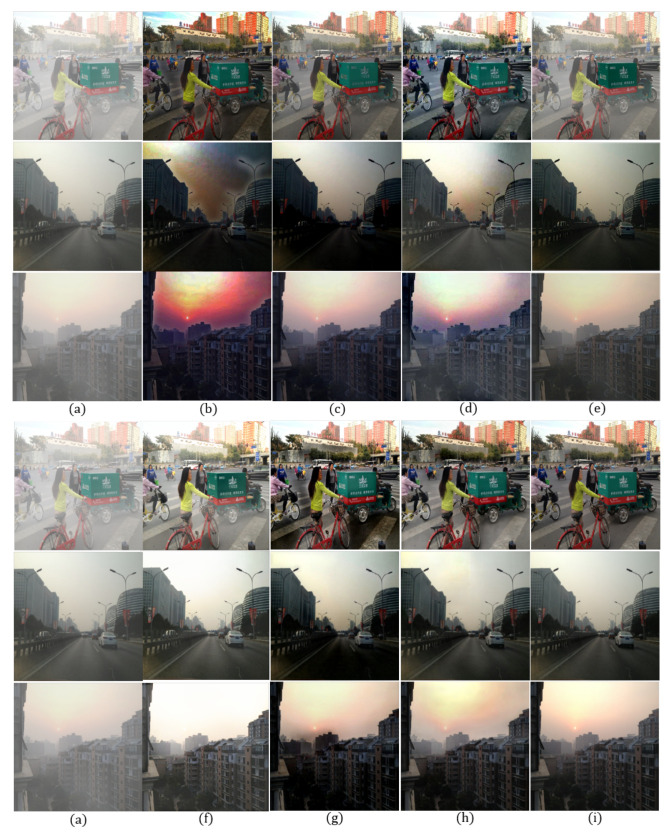
Comparison of dehazing results using synthetic outdoor hazy images [35] for qualitative evaluation: (**a**) hazy input image, (**b**) DCP [9], (**c**) CAP [10], (**d**) RRO [4], (**e**) AOD [37], (**f**) DCPDN [17], (**g**) GFN [18], (**h**) the proposed method, and (**i**) clean image.

**Figure 6:**
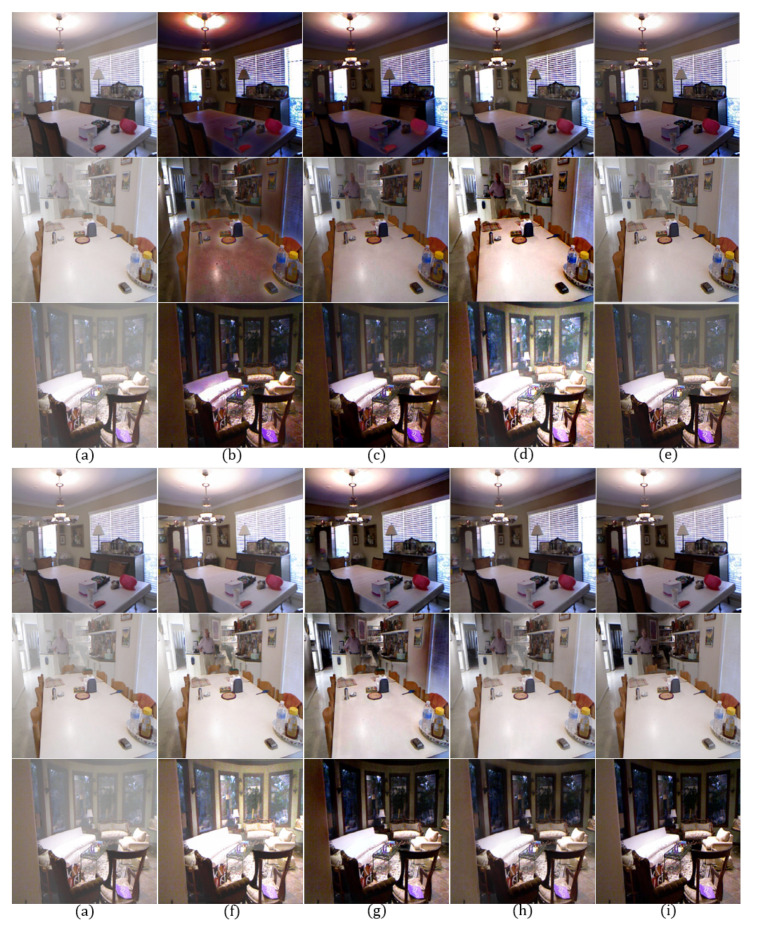
Comparison of dehazing results using synthetic indoor hazy images [35] for qualitative evaluation: (**a**) hazy input image, (**b**) DCP [9], (**c**) CAP [10], (**d**) RRO [4], (**e**) AOD [37], (**f**) DCPDN [17], (**g**) GFN [18], (**h**) the proposed method, and (**i**) clean image.

In order to evaluate the qualitative performance, we show dehazing results using different methods for both outdoor and indoor images as shown in Figure 5 and Figure 6.

Figure 5b shows an increased contrast, but the road region is over-enhanced due to an inaccurately estimated transmission map, resulting in color distortion and halo effect near strong edges. Figure 5c also shows a color distortion problem and remaining haze. Figure 5d shows completely removed haze at the cost of over-enhancement in the sky region. In Figure 5e, haze in all regions is not completely removed. Figure 5f exhibits an over-saturation in the sky region and an increased brightness. Figure 5g shows halo effect around strong edges and over-enhancement problem. On the other hand, the proposed method provides high-quality images without the aforementioned problems.

The contrast of the Figure 6b,c is significantly increased, but their colors were distorted due to the incorrect transmission map estimation. In Figure 6d, the contrast is increased, but the haze is not completely removed. Figure 6e can preserve the original color, but the image is turbid. Figure 6f is brighter than normal and exhibits over-saturation around the chandelier. In Figure 6g, haze is removed well, but there are still halo artifacts near the strong edges. On the other hand, the proposed method provides a clean, high-quality image without color distortion or over-saturation even in an indoor environment.

### 4.3. Ablation Study

**Figure 7:**
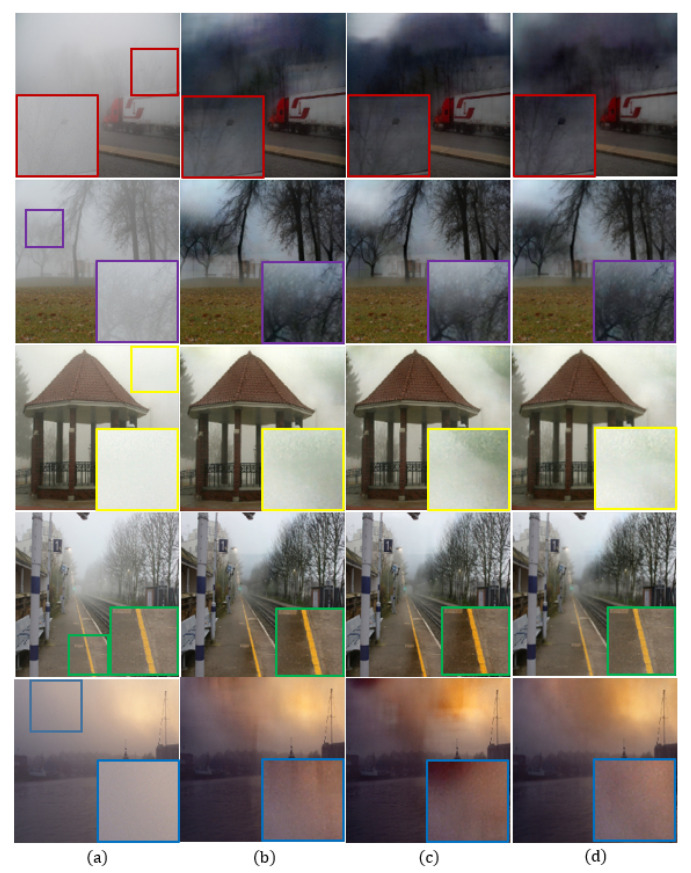
The effect of different loss functions in the proposed model using FADE [36]: (**a**) hazy input image, (**b**) only $L_{l_{1}}$, (**c**) $L_{l_{1}}+L_{vgg}$, and (**d**) the proposed method.

We present results of the ablation study by comparing results using: (i) only $L_{l_{1}}$, (ii) $L_{l_{1}}+L_{vgg}$, and (iii) the complete version of the proposed method as shown in Figure 7. We also present the quantitative evaluation of the same ablation study with respect to different numbers of epochs as shown in Table 8. The experiments were performed using 500 non-reference real-world images without clean image pairs of FADE [36], and computes natural image quality evaluator (NIQE) [40]. A lower NIQE score indicates a relatively higher quality image.

As shown in the enlarged purple box in the second row of Figure 7b, the dehazing result only exhibits undesired artifacts between the haze and tree regions, which results in an unnatural image. Figure 7c still has undesired artifacts and over-enhancement in the hazy region. Furthermore, color distortion occurs near the yellow line in the enlarged green box in the fourth row. On the other hand, the proposed method provides high-quality images without undesired artifacts.

### 4.4. Subjective Quality Comparison

Both quantitative and qualitative evaluations were conducted with the methods compared in Section 4.1 and Section 4.2 to confirm that the proposed method provides high-quality images even in the real-world. To evaluate the non-reference image quality, the averages of FADE [36] 500 images were measured by NIQE and entropy and are given in Table 9.

**Figure 8:**
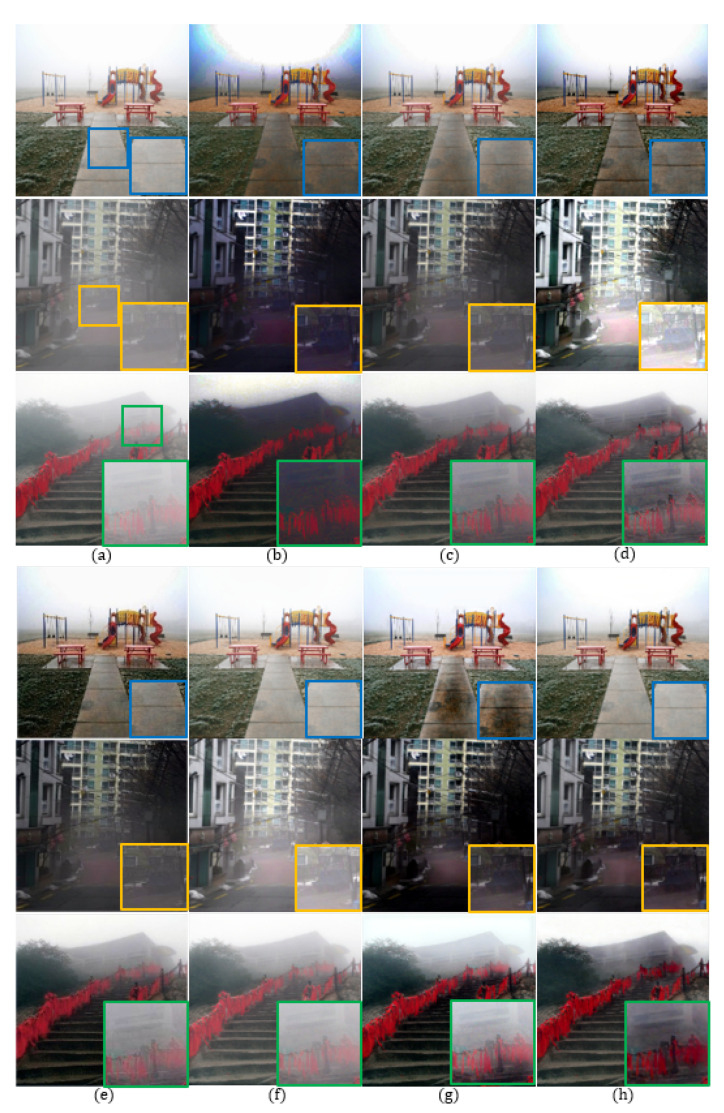
Subjective comparison of different dehazing methods using FADE [36]: (**a**) hazy input image, (**b**) DCP [9], (**c**) CAP [10], (**d**) RRO [4], (**e**) AOD [37], (**f**) DCPDN [17], (**g**) GFN [18], and (**h**) the proposed method.

As shown in enlarged blue boxes in Figure 8, existing methods result in over-enhanced roads, whereas the proposed method successfully removes the haze in the entire image without an over-enhanced problem. As shown in enlarged yellow boxes in Figure 8, the DCP generates color distortion in Figure 8b, and the RRO generates over-saturation in Figure 8d. On the other hand, the proposed reconstructs the shape of the vehicle to become visible in Figure 8h. As shown in enlarged green boxes in Figure 8, the haze is not removed well, whereas the proposed method makes the person clearly visible.

In Table 9, the best score is marked in red, and the second best score is marked in blue. The proposed method gives the highest NIQE score and the second best entropy score with little difference from the best score. Based on these results, the proposed method clearly outperforms existing methods in the sense of both qualitative and quantitative evaluations. In Table 10, the execution times of the proposed method and learning-based methods are compared.The AOD method was faster, but the proposed method not only has better scores than AOD in terms of both NIQE and entropy, but also was faster than DCPDN and GFN. In addition, when the proposed method implemented under a GPU environment, the processing times can be reduced over 100 times.

## 5. Conclusions

If the atmospheric scattering model is inaccurately estimated, the resulting images become degraded. To solve this problem, we proposed a residual-based dehazing network without estimating the atmospheric scattering model, where a gate block and local residual blocks are applied to widen the receptive field without a bottleneck problem, and global residual operation is applied for robust training. The combined structures were designed to construct a robust generator while solving the problem arising from each structure. The proposed model is trained by minimizing the combined VGG16 loss, mean absolution error loss. Furthermore, LSGAN-based learning was applied to acquire robust results for a real image. The discriminator reduces the statistical difference between dehazed and clean images to reduce the statistical divergence. In order to prove the effectiveness of key elements in the proposed method, we conducted an ablation study with an in-depth analysis in Section 4.1. We compared the dehazing performance of the proposed method with state-of-the-art methods for both synthetic and real-world haze images in Section 4 to show that the proposed method performs best in the sense of metrics such as PSNR, SSIM, CIEDE2000, MSE, NIQE, and second in sense of entropy. This shows that the proposed model is a robust haze removal network without estimation of the atmospheric scattering model. In the future research, we will improve the proposed networks to remove dense haze.

## Figures and Tables

**Table 1:** Details of encoder and residual blocks.

Encoder					Residual Blocks				
**Var.**	**Type**	**I/O Filters**	**Size/Stride**	**Acti.**	**Var.**	**Type**	**I/O Filters**	**Size/Stride**	**Acti.**
Input: *I*					Input: $E_{4},R_{1},R_{2},R_{3},R_{4}$				
$E_{1}$	Conv	3/64	3/1	Relu	$E_{5n}$	Conv	128/64	3/1	Relu
$E_{2}$	Conv	64/64	3/1	Relu	$E_{5n+1}$	Conv	64/64	3/1	Relu
$E_{3}$	Conv	64/128	3/2	Relu	$E_{5n+2}$	Conv	64/64	3/1	Relu
$E_{4}$	Conv	128/128	3/2	Relu	$E_{5n+3}$	Conv	64/64	3/1	Relu
					$E_{5n+4}$	Conv	64/64	3/1	
					$R_{n}$	Sum			Relu
Output: $E_{4}$					Output: $R_{1},R_{2},R_{3},R_{4},R_{5}$				

**Table 2:** Details of gate block and decoder.

Gate Block					Decoder				
**Var.**	**Type**	**I/O Filters**	**Size/Stride**	**Acti.**	**Var.**	**Type**	**I/O Filters**	**Size/Stride**	**Acti.**
Input: $Concat(R_{1},R_{2},R_{3},R_{4},R_{5})$					Input: $G_{f_{total}}$				
$G_{f_{1,2,\cdots ,5}}$	Conv	320/5	3/1		${↑}_{2}$	2x			
					$E_{31}$	Conv	64/64	3/1	
					${↑}_{2}$	2x			
					$E_{32}$	Conv	64/64	3/1	
					$E_{33}$	Conv	64/64	3/1	Relu
					$E_{34}$	Conv	64/64	3/1	
					$E_{34}+I$	Sum			Relu
Output: $G_{f_{total}}=\sum_{k=1}^{5}G_{f_{k}}\circ R_{k}$					Output: $G\left(I\right)$				

**Table 3:** Details of discriminator.

Discriminator				
**Var.**	**Type**	**I/O Filters**	**Size/Stride**	**Acti.**
Input: $G\left(I\right)$ or *J*				
$C_{1}$	Conv	3/48	3/1	
$BN_{1}$	Batch norm	48		Lrelu
$C_{2}$	Conv	48/96	3/1	
$BN_{2}$	Batch norm	96		Lrelu
$C_{3}$	Conv	96/192	3/1	
$BN_{3}$	Batch norm	192		Lrelu
$C_{4}$	Conv	192/1	3/1	Sigmoid
Output: S1				

**Table 4:** Comparative SSIM results of dehazing methods on SOTS. Best score is marked in red.

SSIM		DCP [9]	CAP [10]	RRO [4]	AOD [37]	DCPDN [17]	GFN [18]	Proposed
Outdoor	Mean	0.8156	0.8159	0.8223	0.8947	0.8649	0.8717	0.9589
	Std	0.0733	0.1171	0.0476	0.0453	0.0743	0.0779	0.0222
	Min	0.6114	0.4142	0.6329	0.6677	0.6134	0.5260	0.8522
	Max	0.9657	0.9879	0.9198	0.9722	0.9834	0.9871	0.9910
Indoor	Mean	0.8431	0.8447	0.8197	0.8362	0.7451	0.9113	0.9377
	Std	0.0563	0.0622	0.0862	0.0779	0.1132	0.0327	0.0273
	Min	0.6775	0.6276	0.5489	0.5529	0.4331	0.8089	0.8569
	Max	0.9458	0.9652	0.9618	0.9610	0.9549	0.9767	0.9852

**Table 5:** Comparative CIEDE2000 results of dehazing methods on SOTS. Best score is marked in red.

CIEDE2000		DCP [9]	CAP [10]	RRO [4]	AOD [37]	DCPDN [17]	GFN [18]	Proposed
Outdoor	Mean	14.0003	9.8399	10.1197	7.7192	11.3252	6.2929	4.2070
	Std	4.9894	3.1774	2.9756	2.5158	4.5708	2.6843	2.1787
	Min	3.6469	2.4560	3.9555	2.4727	2.0515	2.3672	1.6188
	Max	33.2482	19.7684	20.3894	17.9665	29.8194	16.8856	15.2767
Indoor	Mean	10.6250	8.2445	10.1597	8.1847	17.5894	5.5170	4.7627
	Std	3.9784	3.0052	3.7726	3.8485	5.4186	1.2776	1.5630
	Min	4.4610	3.5705	4.6058	3.0409	6.6099	2.8880	1.7230
	Max	26.9328	20.7123	24.8660	24.3492	34.4222	13.7701	9.8903

**Table 6:** Comparative PSNR results of dehazing methods on SOTS. Best score is marked in red.

PSNR		DCP [9]	CAP [10]	RRO [4]	AOD [37]	DCPDN [17]	GFN [18]	Proposed
Outdoor	Mean	14.8567	18.4366	18.6245	19.6552	17.5784	22.1102	25.5830
	Std	2.9009	3.1846	2.5864	2.4030	3.3812	4.1891	4.2117
	Min	8.0599	12.7874	11.7237	12.8299	8.8716	12.5997	13.6739
	Max	26.1906	34.5381	26.3478	27.4443	31.5347	33.1781	34.2140
Indoor	Mean	17.0254	19.1585	18.0264	18.8528	13.0895	22.6664	23.6014
	Std	2.7620	2.5793	3.0466	3.2735	2.7285	2.6877	2.9066
	Min	9.4716	10.8411	9.0158	10.3277	6.9896	13.1246	16.2261
	Max	24.0988	25.3788	23.5649	26.6223	20.6942	29.6524	31.6312

**Table 7:** Comparative MSE results of dehazing methods on SOTS. Best score is marked in red.

MSE(%)		DCP [9]	CAP [10]	RRO [4]	AOD [37]	DCPDN [17]	GFN [18]	Proposed
Outdoor	Mean	4.14	1.83	1.77	1.27	2.29	0.98	0.49
	Std	2.42	1.08	1.08	0.69	1.74	0.94	0.67
	Min	0.28	0.06	0.26	0.20	0.07	0.05	0.04
	Max	15.06	5.54	6.78	4.8	12.83	5.9	4.96
Indoor	Mean	2.53	1.51	2.15	1.78	5.82	0.69	0.56
	Std	1.71	1.02	1.96	1.57	3.44	0.49	0.40
	Min	0.49	0.29	0.46	0.30	0.84	0.13	0.07
	Max	11.38	8.66	13.14	9.26	20.14	4.85	2.41

**Table 8:** NIQE [40] measurement results on the effect of loss functions.

Methods	40	80	120	160	200
$L_{l_{1}}$	3.6836	3.6145	3.5732	3.5523	3.5656
$L_{l_{1}}+L_{vgg}$	3.6557	3.6461	3.6044	3.5494	3.5125
Proposed method	3.4893	3.4686	3.451	3.4931	3.5039

**Table 9:** Comparative results of dehazing methods on FADE [36]. Best score is marked in red, and the second best score is marked in blue.

Metric	DCP [9]	CAP [10]	RRO [4]	AOD [37]	DCPDN [17]	GFN [18]	Proposed
NIQE [40]	4.0023	4.1433	4.4429	4.0059	4.3767	4.2806	3.5039
Entropy [36]	6.8954	7.1554	7.327	7.0785	7.1664	7.2664	7.2745

**Table 10:** Comparative execution times of the dehazing methods (seconds).

Resolution	AOD (cpu) [37]	DCPDN (cpu) [17]	GFN (cpu) [18]	Proposed (cpu)	Proposed (gpu)
256×256	0.14	n/a	10.12	2.23	0.01
512×512	0.49	12.56	40.51	8.99	0.04
1024×1024	1.9045	n/a	163.62	38.34	0.17
Framework	Pytorch	Pytorch	Matlab-caffe	Pytorch	Pytorch
